# Binding of Glycoprotein Srr1 of *Streptococcus agalactiae* to Fibrinogen Promotes Attachment to Brain Endothelium and the Development of Meningitis

**DOI:** 10.1371/journal.ppat.1002947

**Published:** 2012-10-04

**Authors:** Ho Seong Seo, Rong Mu, Brandon J. Kim, Kelly S. Doran, Paul M. Sullam

**Affiliations:** 1 Division of Infectious Diseases, Veterans Affairs Medical Center and the University of California, San Francisco, California, United States of America; 2 Department of Biology and Center for Microbial Sciences, San Diego State University, San Diego, California, United States of America; 3 Department of Pediatrics, University of California at San Diego, School of Medicine, La Jolla, California, United States of America; Children's Hospital Boston, United States of America

## Abstract

The serine-rich repeat glycoprotein Srr1 of *Streptococcus agalactiae* (GBS) is thought to be an important adhesin for the pathogenesis of meningitis. Although expression of Srr1 is associated with increased binding to human brain microvascular endothelial cells (hBMEC), the molecular basis for this interaction is not well defined. We now demonstrate that Srr1 contributes to GBS attachment to hBMEC via the direct interaction of its binding region (BR) with human fibrinogen. When assessed by Far Western blotting, Srr1 was the only protein in GBS extracts that bound fibrinogen. Studies using recombinant Srr1-BR and purified fibrinogen *in vitro* confirmed a direct protein-protein interaction. Srr1-BR binding was localized to amino acids 283–410 of the fibrinogen Aα chain. Structural predictions indicated that the conformation of Srr1-BR is likely to resemble that of SdrG and other related staphylococcal proteins that bind to fibrinogen through a “dock, lock, and latch” mechanism (DLL). Deletion of the predicted latch domain of Srr1-BR abolished the interaction of the BR with fibrinogen. In addition, a mutant GBS strain lacking the latch domain exhibited reduced binding to hBMEC, and was significantly attenuated in an *in vivo* model of meningitis. These results indicate that Srr1 can bind fibrinogen directly likely through a DLL mechanism, which has not been described for other streptococcal adhesins. This interaction was important for the pathogenesis of GBS central nervous system invasion and subsequent disease progression.

## Author Summary

*Streptococcus agalactiae* (Group B *streptococcus*, GBS) is a leading cause of meningitis in newborns and infants. This life-threatening infection of the brain and surrounding tissues continues to result in a high incidence of morbidity and mortality, despite antibiotic therapy. A key factor in disease production is the ability of this organism to invade the central nervous system, via the bloodstream. We now report that a GBS surface protein called Srr1 binds fibrinogen, a major protein in human blood. This interaction enhances the attachment of GBS to brain vascular endothelial cells, and contributes to the development of meningitis. A mutation in Srr1 that specifically disrupted binding to fibrinogen significantly reduced GBS attachment to brain endothelium, and markedly reduced virulence in an *in vivo* model of GBS disease. These studies have identified a new mechanism by which Srr1 contributes to GBS invasion of the central nervous system and may provide a basis for novel therapies targeting Srr1 binding.

## Introduction

**Figure 1:**
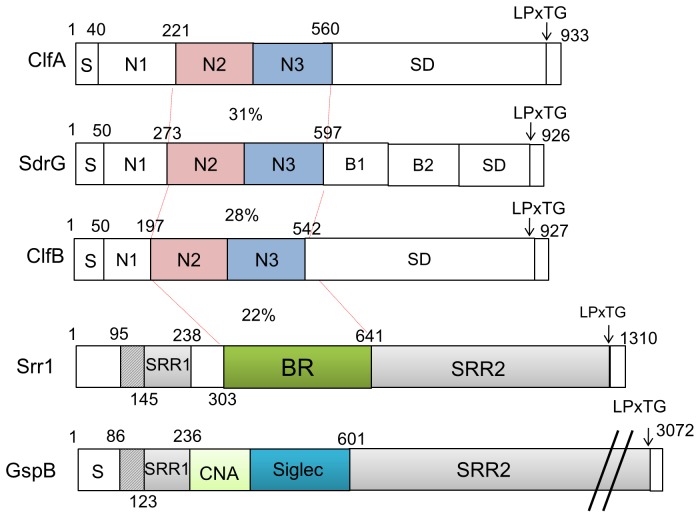
Schematic diagram of three staphylococcal fibrinogen binding proteins (ClfA, SdrG and ClfB) and the serine rich repeat proteins Srr1 and GspB. Level of identity (%) between regions is indicated. *S: signal sequence; N1, N2, and N3: DEv-IgG domains; B1 and B2, repeats of unknown function; SD: serine and aspartic acid rich region; SRR1 and SRR2: serine rich regions; CNA: IgG fold domain; Siglec: sialic acid binding domain; LPxTG: cell wall anchoring motif.

The serine-rich repeat (SRR) glycoproteins are a large and diverse family of adhesins found in Gram-positive bacteria [1], [2]. Each SRR protein is encoded within a large locus that also contains genes encoding proteins responsible for glycosylating the SRR protein, as well as an accessory Sec system that is dedicated to the export of the adhesin. The SRR proteins have a highly conserved domain organization, including a long and specialized signal sequence, two extensive serine-rich repeat regions that undergo glycosylation, and a typical LPXTG cell wall anchoring motif [3], [4]. The N-termini also contain a binding region that varies considerably, both in terms of structure and adherence properties (Figure 1).

Among the best-characterized is GspB of *Streptococcus gordonii*, which binds human platelets through its interaction with sialyl-T antigen on the platelet receptor GPIbα [2], [5]. This appears to be an important event in the pathogenesis of infective endocarditis, since disruption of Siglec-mediated binding results in reduced virulence, as measured by an animal model of endocardial infection [3], [4]. A number of other SRR proteins have been shown to contribute to virulence, including SraP of *Staphylococcus aureus*, PsrP of *Streptococcus pneumoniae*, and the two SRR proteins (Srr1 and Srr2) of GBS [6]–[11]. However, the molecular basis for binding by these other adhesins is less defined. Their binding regions have no homology to that of GspB, indicating that they are not Siglec-like adhesins. Although SraP mediates binding to platelets, the receptor for this SRR protein has not been identified [6]. PsrP binds cytokeratin 10 *in vitro*, which appears to be important for binding to pulmonary epithelial cells and subsequent pneumonia [12].

Expression of Srr1 or Srr2 by GBS has been shown to contribute to virulence in models of meningitis [7], [8]. Srr1 mediates binding to several types of human epithelial cell lines, as well as human brain microvascular endothelial cells (hBMEC) [7], [13]. Binding of these cells appears to be important for both colonization and invasion. *In vitro* studies have indicated that one ligand for Srr1 is human keratin 4, which may facilitate attachment to cervical, vaginal, and pharyngeal cells [13], [14]. We now report, however, that Srr1 also binds human fibrinogen directly through its interaction with the Aα chain of the heteromultimeric protein. This interaction mediates the binding of GBS both to fibrinogen and to hBMEC, and appears to be important for virulence in the setting of meningitis.

## Results

### Srr1 mediates GBS binding

**Figure 2:**
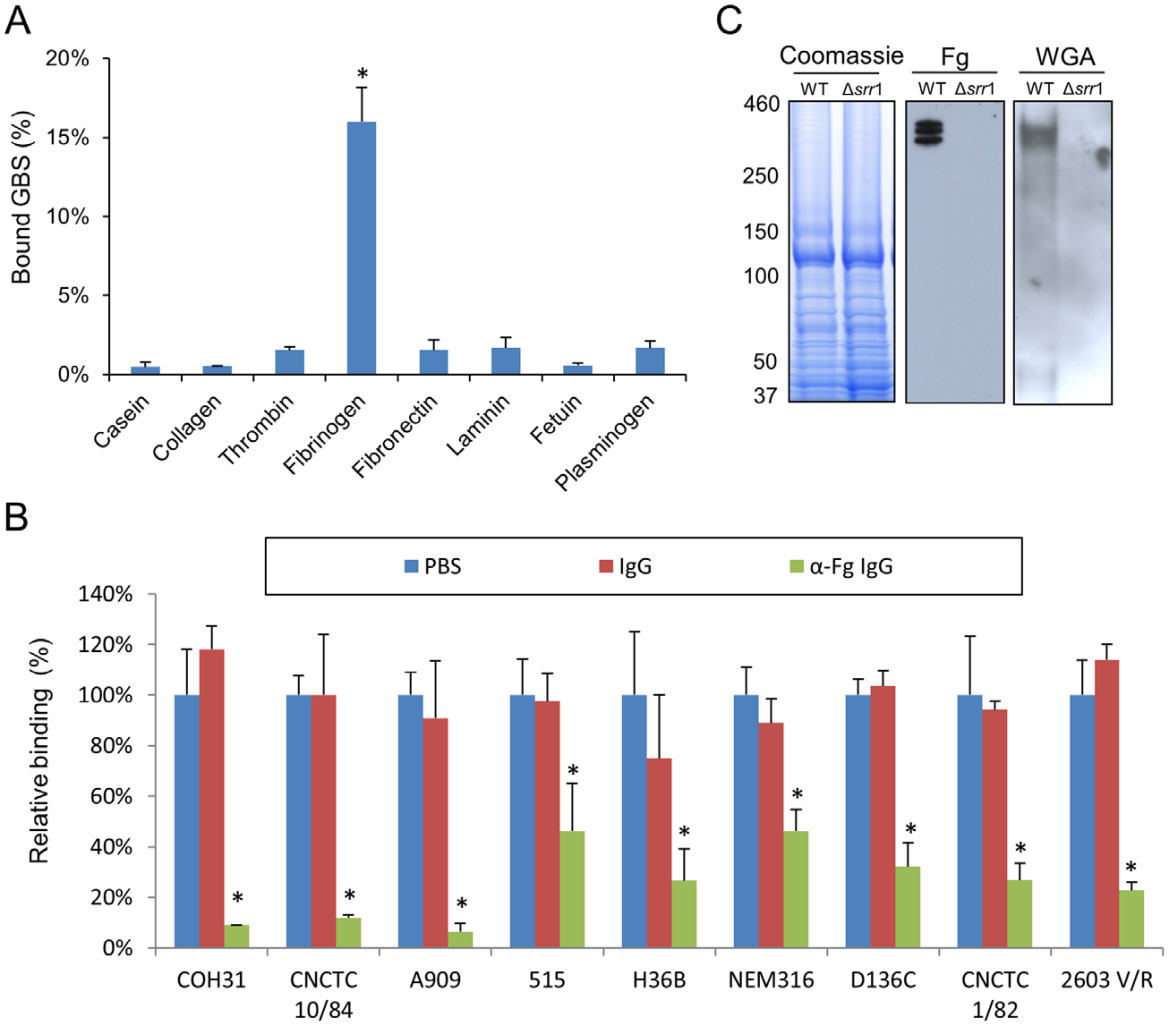
Binding of GBS to fibrinogen. (A) GBS strain COH31 strain was incubated with wells pretreated with collagen, thrombin, fibrinogen, fibronectin, laminin, fetuin, or plasminogen (0.1 µM per well). Values represent mean ± S.D. of the percent of inoculum bound. (B) Immobilized fibrinogen was preincubated with rabbit anti-fibrinogen IgG (100 µg/ml) or rabbit IgG (100 µg/ml) prior to testing for binding by GBS. Unbound IgG was removed by washing and GBS binding was assessed. Values represent percent of GBS binding as compared with untreated fibrinogen. (C) Far Western blotting of cell wall proteins from strain COH1 (WT) and PS954 (Δ*srr1*). Left panel shows SDS-PAGE of cell wall preparations. Proteins were transferred to nitrocellulose and probed with purified fibrinogen (fibrinogen; 10 µg/ml, middle panel) or biotinylated WGA lectin (0.2 µg/ml, right panel). Bound fibrinogen and lectin were detected with anti-fibrinogen IgG or HRP conjugated streptavidin, respectively. * = P<0.01.

We first measured the adherence of GBS strain COH31 (a serotype III clinical isolate) to a variety of host plasma and matrix proteins. As shown in Fig. 2A, GBS adhered to immobilized human fibrinogen at levels (mean: 16±2.8% of inoculum) that were significantly higher than those seen to with the negative control, casein (<1%). Low levels of binding (<2%) were observed with thrombin, fibronectin, laminin, plasminogen, collagen IV, and fetuin. Binding was significantly inhibited by pretreatment of immobilized fibrinogen with anti-fibrinogen IgG, indicating that the interaction between GBS and fibrinogen was specific (Figure 2B). We also examined eight additional GBS isolates, representing a range of capsular types, all of which were found to bind immobilized fibrinogen. As was seen with the COH31 strain, binding of all GBS strains tested was significantly reduced during treatment with IgG specific for fibrinogen. These data indicate that GBS can adhere specifically to immobilized fibrinogen and adherence to fibrinogen is a general property of GBS.

To better characterize the GBS surface components responsible for fibrinogen interaction, we examined the binding of soluble human fibrinogen to GBS cell wall proteins by Far Western blotting. Although the GBS cell wall extracts contained numerous proteins (Figure 2C, left panel), fibrinogen binding was restricted to a group of high MW bands (300–400 kDa) (middle panel). Probing the membranes with WGA revealed binding of the lectin to one or more proteins of similar size, indicating that they were glycosylated (right panel). Since the serine-rich repeat protein Srr1 of GBS is a high MW glycoprotein, we next assessed the impact of deleting *srr1* on WGA and fibrinogen binding. When cell wall extracts of COH31Δ*srr1* (PS954) were probed with WGA or fibrinogen, no binding was observed, confirming that the glycoprotein bound by fibrinogen was Srr1.

**Figure 3:**
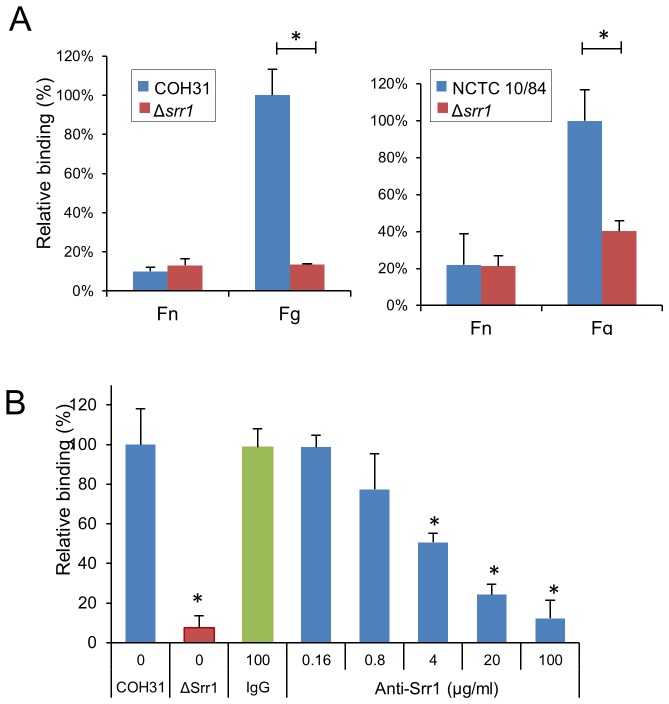
GBS binding to fibrinogen is mediated by Srr1. (A) GBS strains COH31 (left) and NCTC 10/84 (right) were compared with their Δ*srr1* variants (Δ*srr1*) for binding to the wells pretreated with fibronectin or fibrinogen (0.1 µM per well). (B) Inhibition of GBS binding to fibrinogen by anti-Srr1 IgG. COH31 strain was co-incubated with rabbit anti-Srr1 IgG or normal rabbit IgG, and relative binding to immobilized with fibrinogen. Values are mean ± S.D. of relative binding, normalized for WT levels of binding to fibrinogen. * = P<0.01.

To examine the impact of Srr1 expression on bacterial binding to fibrinogen, we tested the ability of GBS strains COH31 and NCTC 10/84, and Δ*srr1* variants to bind to immobilized fibrinogen. As shown in Figure 3A, deletion of *srr1* markedly reduced GBS binding to fibrinogen. Similar results were observed with additional GBS strains H36B and 515 (data not shown). To confirm the role of Srr1 expression in fibrinogen binding by GBS, we next assessed whether binding by COH31 and NCTC 10/84 to fibrinogen was inhibited by rabbit anti-Srr1 IgG (Figure 3B and Figure S1). In control studies, co-incubation of either strain with rabbit IgG had no effect on fibrinogen binding. In contrast, co-incubation of GBS with anti-Srr1 IgG significantly reduced binding to fibrinogen. The level of inhibition was concentration-dependent, with 100 µg/ml of anti-Srr1 IgG being sufficient to reduce WT GBS binding to levels comparable to those seen with GBSΔ*srr*1. Complementation of the *srr1* mutation *in trans* restored fibrinogen binding by NCTC 10/84 Δ*srr1* (Figure S2), thereby demonstrating that the loss of binding observed with *srr1* disruption was not due to polar or pleiotropic effects. These results indicate that GBS binding to immobilized fibrinogen is mediated by the surface expressed Srr1 protein.

**Figure 4:**
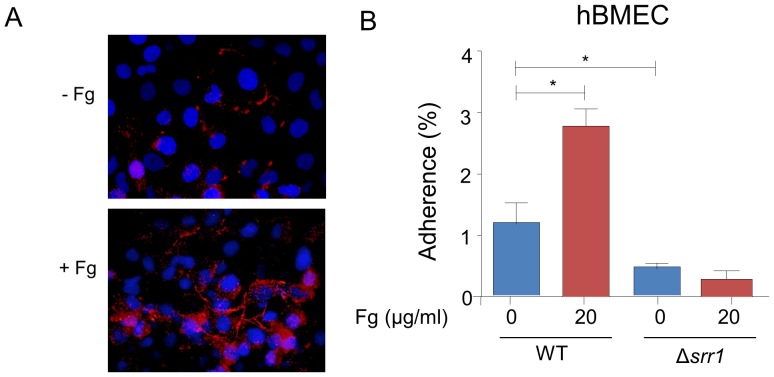
GBS adherence to hBMEC is mediated by the interaction of Srr1 and fibrinogen. (A) Fibrinogen on the surface of hBMEC pretreated with or without exogenous fibrinogen (Fg; 20 µg/ml). Nuclei were stained with DAPI (blue) and fibrinogen was detected with anti-fibrinogen IgG, followed by Alexa Fluor 488 conjugated anti-rabbit IgG (red). (B) NCTC 10/84 (WT) or PS2645 (Δ*srr1*) incubated with hBMEC, with or without fibrinogen pretreatment (20 µg/ml). Unbound bacteria were washed out and bound bacteria were counted. Values represent percent (mean ± S.D.) of total GBS inoculum bound to the monolayers. * = P<0.01.

The attachment of GBS to human brain microvascular endothelial cells (hBMEC) is thought to be important for the invasion of the central nervous system by this organism [15]–[17]. Previous studies indicate that binding of GBS to brain endothelium is mediated by Srr1 [7]. To assess whether fibrinogen contributed to this interaction, we assessed the role of fibrinogen in Srr1-mediated binding of GBS to hBMEC. Fibrinogen was detectable on the surface of washed hBMEC, as measured by immunofluorescence microscopy (Figure 4A). Exposure of the cells to exogenous human fibrinogen (20 µg/ml), markedly increased the amount of the protein on the cell surface, indicating that hBMEC are capable of binding fibrinogen. Strain NCTC10/84 and an isogenic Δ*srr1* variant (PS2645) were incubated with hBMEC in tissue culture wells. After 30 min, WT GBS efficiently adhered to these cells, whereas the Δ*srr1* mutant was significantly reduced in binding (*p*<0.01) (Figure 4B). Preincubation of bacteria with purified human fibrinogen (20 µg/ml) enhanced the binding of the WT strain to hBMEC, but had no effect on binding of the Δ*srr1* mutant strain.

### Biochemical characterization of the binding domain of Srr1

**Figure 5:**
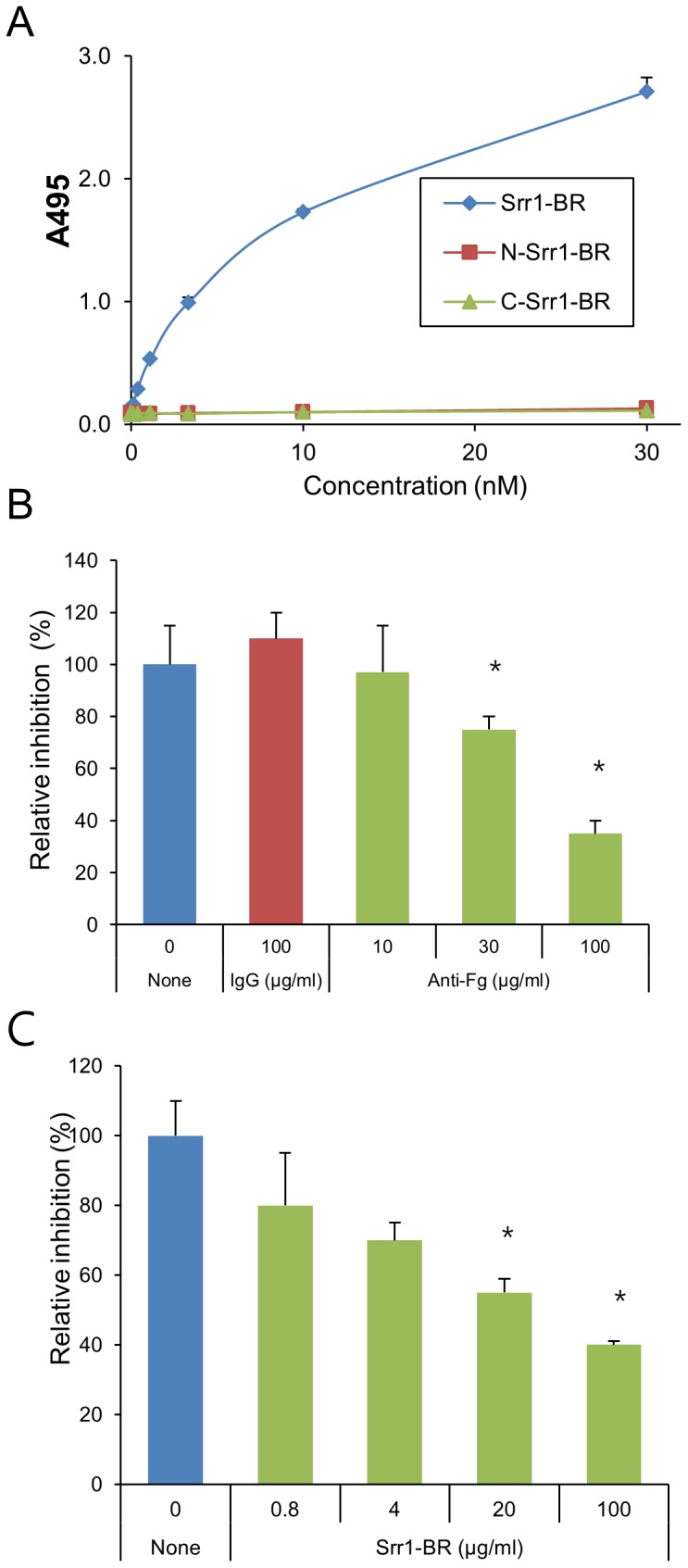
Interaction of the binding region (BR) of Srr1 with fibrinogen. (A) Binding of purified Srr1-BR, N terminal Srr1-BR, or C-terminal Srr1-BR protein (_FLAG_Srr1-BR, _FLAG_N-Srr1-BR, _FLAG_C-Srr1-BR) to immobilized fibrinogen (0.1 µM). Bound proteins were detected with anti-FLAG antibody. (B) Inhibition of _FLAG_Srr1-BR binding to immobilized fibrinogen with anti-fibrinogen IgG. Immobilized fibrinogen was preincubated with the indicated concentration of anti-fibrinogen antibody, and then tested for binding by _FLAG_Srr1-BR (5 µg/ml). Bound proteins were detected with anti-FLAG IgG. Normal rabbit IgG served as a control. (C) Inhibition of _FLAG_Srr1-BR binding to fibrinogen by unlabeled Srr1-BR. Immobilized fibrinogen was coincubated with the indicated concentrations of _FLAG_Srr1-BR (5 µg/ml) or unlabeled Srr1-BR. Bound proteins were detected with anti-FLAG antibody. Values represent percent of _FLAG_Srr1-BR binding to the wells treated with fibrinogen. Bars indicate the means (± S.D.). * = P<0.01.

The ligand binding site of the SRR proteins characterized to date has been localized to the region bridging the two serine-rich repeat domains (Figure 1) [1]–[3], [6], [9]. To confirm that the putative binding region of Srr1 (Srr1-BR) interacts with fibrinogen, we assessed the binding of the purified FLAG tagged binding region (_FLAG_Srr1-BR) with fibrinogen. In control studies, no significant binding by _FLAG_Srr1-BR to immobilized casein blocking regent was detected. In contrast, _FLAG_Srr1-BR showed significant binding to fibrinogen, which increased in direct proportion to the amount of protein applied (Figure 5A). No fibrinogen binding activity was detected by either the N-terminal of Srr1-BR (AA303–479) or C-terminus (AA480–641) alone, indicating that entire region is required. To determine the apparent *K_D_* for the binding of _FLAG_Srr1-BR to fibrinogen, we analyzed data from six independent ELISA-based binding assays, as described previously. The calculated mean *K*_D_ was 7.51×10^−8^, which is within the range reported for staphylococcal fibrinogen binding proteins [18]. To validate these findings, we also examined the inhibition of this interaction with either anti-fibrinogen IgG or unlabeled Srr1-BR (Figure 5C and D). When immobilized fibrinogen was pretreated with anti-fibrinogen IgG, the binding of _FLAG_Srr1-BR to the protein was subsequently reduced (Figure 5C). In addition, when _FLAG_Srr1-BR was co-incubated with unlabeled (non-tagged) Srr1-BR, subsequent binding was effectively blocked (Figure 5D). These findings indicate that the fibrinogen binding domain of the Srr1 is indeed located in the binding region (AA 303–641).

### Identification of the binding site for Srr1-BR

**Figure 6:**
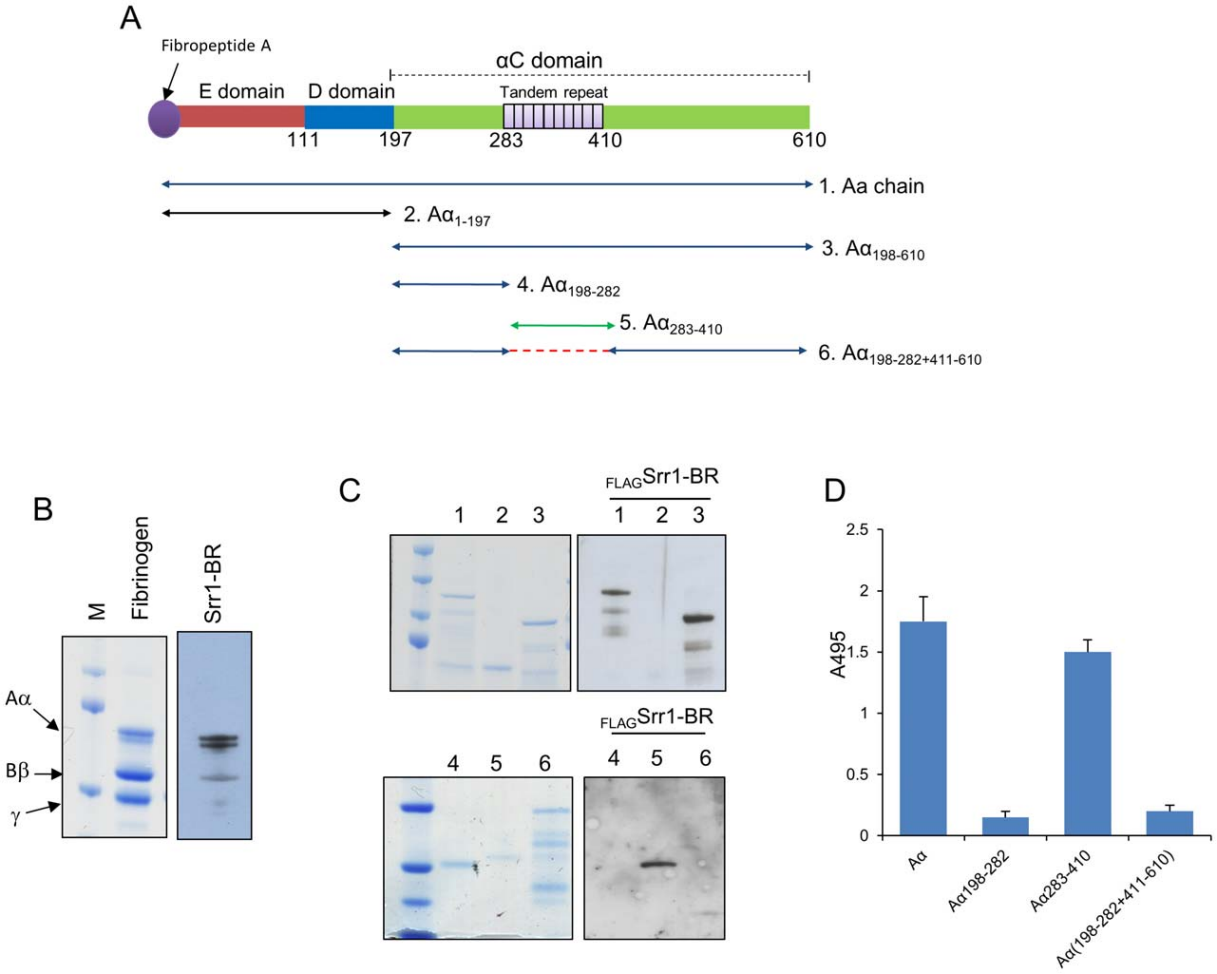
Srr1-BR binding to αC domain of the fibrinogen Aα chain. (A) Schematic of fibrinogen Aα organization. The 10 tandem repeating units of the αC domain are represented by the purple bars. Arrows #1–6 indicate the truncated forms of Aα chain tested. (B) Binding of _FLAG_Srr1-BR to fibrinogen Aα chain. Purified human fibrinogen was separated by SDS-PAGE and stained with Coomassie blue (left) or transferred to nitrocellulose and probed with _FLAG_Srr1-BR (5 µg/ml; right). (C) Recombinant MalE-Aα variants were separated by SDS-PAGE and stained with Coomassie blue (left) or transferred to nitrocellulose and probed with _FLAG_Srr1-BR (right). (D) _FLAG_Srr1-BR (2 µg/ml) was incubated with immobilized recombinant MalE-Aα variants. Binding was detected by ELISA with anti-FLAG antibody. Bars indicate the means (± S.D.).

We next sought to characterize the region within fibrinogen responsible for Srr1-BR binding. Fibrinogen is a complex protein consisting of two subunits, each containing three polypeptide chains (Aα, Bβ and γ). When separated by SDS-PAGE under reducing conditions, fibrinogen appeared as three bands corresponding to the Aα, Bβ, and γ chains (Aα = 63.5 kDa, Bβ = 56 kDa, γ = 47 kDa) having the expected masses (Figure 6B). When transferred to nitrocellulose and probed with purified _FLAG_Srr1-BR, the Aα chain was readily detected, with low levels of binding seen to the Bβ and γ chains (Figure 6B). We also assessed the binding of Srr1-BR to recombinant forms of each chain, expressed as MalE fusion proteins. In this case, _FLAG_Srr1-BR was found to bind the MalE:Aα chain, while no binding was seen to the MalE:Bβ and MalE:γ chains (Figure S3).

We next sought to identify the domains within the Aα chain bound by Srr1-BR, by examining the binding of Srr1-BR to a series of recombinant Aα chain truncates (Figure 6A and 6C). Far Western blot analysis showed that binding of _FLAG_Srr1-BR was localized to subdomains containing residues 283–410, which correspond to the tandem repeat region of the Aα chain (Figure 6C). To confirm that this region was the Srr1-BR binding site, we assessed by ELISA the interaction of _FLAG_Srr1-BR with the immobilized fibrinogen Aα subdomains (Fig. 6D). As was observed with the Far Western analysis, we found no significant binding of _FLAG_Srr1-BR to immobilized MalE:Aα_198–282_ or MalE:Aα_(198–282+411–610)_. However, _FLAG_Srr1-BR exhibited levels of binding to MalE:Aα_283–410_ that were comparable to recombinant full length Aα chain (MalE:Aα_1–610_), indicating that the Srr1-BR binding site is indeed the 13 AA tandem repeat region within the Aα chain of fibrinogen.

**Figure 7:**
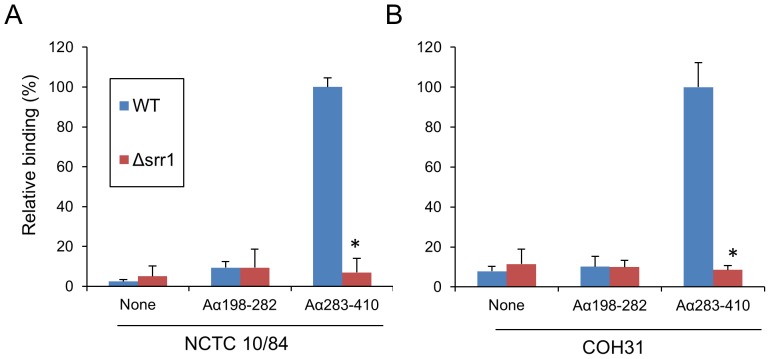
GBS binding to fibrinogen Aα variants. GBS strains NCTC 10/84 (A) and COH31 (B) were compared with their Δ*srr1* variants (Δ*srr1*) for binding to MalE:Aα_198–282_ or MalE:Aα_283–410_. Values are mean ± S.D. percent of WT GBS binding to immobilized MalE:Aα_283–410_. * = P<0.01.

Next we examined whether fibrinogen binding by GBS was mediated by the interaction of Srr1-BR with Aα_283–410_. GBS strains COH31 and NCTC 10/84, and their respective Δ*srr*1 mutants (PS954 and PS2645) were incubated with either immobilized MalE:Aα_283–410_ or MalE:Aα_198–282_ (Fig. 7A and B). The Δ*srr*1 mutant strains exhibited low levels of binding to both Aα chain truncates. In contrast, WT GBS strains had high levels of binding to MalE:Aα_283–410_, as compared with MalE:Aα_198–282_. In addition, we found that GBS binding to immobilized fibrinogen was subsequently reduced during co-incubation with MalE:Aα_283–410_ (Figure S4), suggesting that Srr1-BR binds fibrinogen specifically within AA 283–410 of the Aα chain, and that this interaction is important for GBS fibrinogen binding.

### Sequence analysis of Srr1-BR

To gain a better understanding of the structural determinants present within the binding region of Srr1, bioinformatic analysis was performed on the predicted binding region sequence (AA 303–641). Interestingly, PSI-BLAST analysis identified this region to be related to the fibrinogen binding domain of the staphylococcal adhesins SdrG and ClfA (sharing 22% and 23% identity respectively). Structure prediction analysis using PHYRE -[19], Swiss-Model [20], and HHPRED [21] algorithms also identified the binding region of Srr1 as having structural similarity to the fibrinogen-binding region of SdrG (HHPred; 100% probability, e = 4.5e−51) and ClfA (HHPred; 100% probability, e value = 9.2e−51) (Fig. S5).

The binding regions of ClfA and SdrG are composed of two domains (N2 and N3) (Figure 1), each of which adopts an IgG-like fold [22]–[24]. This domain architecture enables fibrinogen binding through a “dock, lock, and latch” mechanism (DLL) [24], in which fibrinogen engages a binding cleft between the N2 and N3 domains. As the ligand “dock”, the flexible C-terminal extension of the N3 domain (the “latch”) changes conformation, so that it “locks” the ligand in place, and forms a β strand complex with the N2 domain [24]. Bacterial adhesins that are structurally related to Clf-Sdr family are able to bind fibrinogen using this mechanism, which appears to represent a general mode of ligand-adhesin binding [24]–[28]. Collectively, our bioinformatic analysis suggests that the binding region of Srr1 structurally resembles the binding region of the Clf-Sdr family proteins (SdrG, ClfA, ClfB) and may have a similar binding mechanism.

### Impact of Srr1 latch domain on GBS adherence

**Figure 8:**
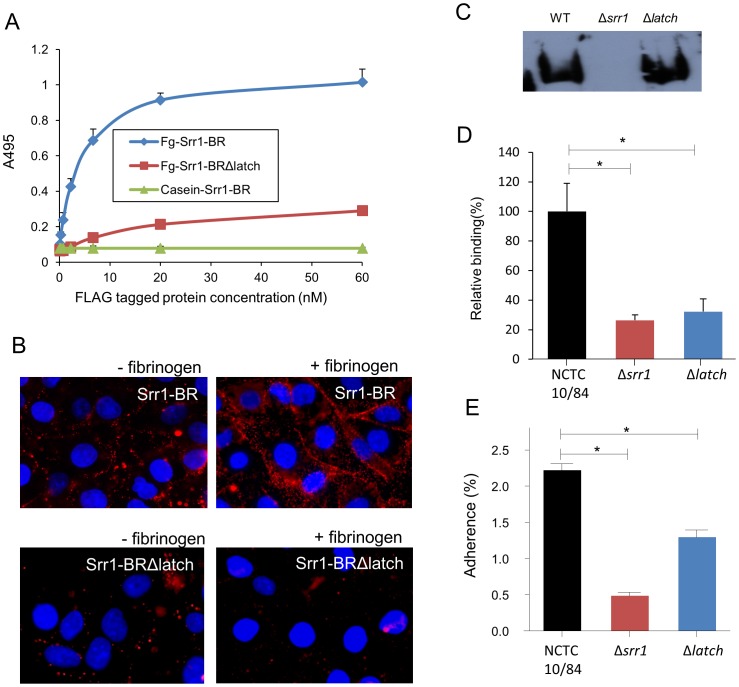
Impact of the Srr1 latch-like domain on GBS binding. (A) Binding of _FLAG_Srr1-BR and _FLAG_Srr1-BRΔlatch proteins to immobilized fibrinogen. Indicated concentration of _FLAG_Srr1-BR and _FLAG_Srr1-BRΔlatch were added to wells coated with fibrinogen or casein blocking reagent. (B) Binding of _FLAG_Srr1-BR and _FLAG_Srr1-BRΔlatch proteins to hBMEC monolayers pretreated with PBS (left panels) or fibrinogen (20 µg/ml, right panels). After washing out unbound proteins, bound proteins were detected with anti-FLAG mAb, followed by Alexa Fluor 488 conjugated anti-mouse IgG (red). Nuclei were stained with DAPI (blue). (C) Expression of Srr1-WT and Srr1Δ*latch* on the cell surface. Isolated cell wall proteins were probed by Western blotting with anti-Srr1 IgG. (D) GBS NCTC 10/84 WT, Δ*srr1* and Δlatch variant binding to immobilized fibrinogen. Values represent percent of WT GBS binding to fibrinogen. (E) GBS NCTC 10/84 WT, Δ*srr1* and Δ*latch* isogenic variant adherence to hBMEC monolayers. * = P<0.01.

Using structure prediction searches (HHPRED) [21], we did not identify a latch-like sequence in C-terminal end of the Srr1-BR. However, a highly homologous TYTFTDYVD-like “latching cleft” sequence between the D1 and E1 strands was identified at AA 412∼420 (TYTWTRYAS) (Figure S5 and Table S3). To investigate whether the C-terminal end of Srr1-BR contained a functional latch-like domain, we generated a variant of Srr1-BR, in which the C-terminal 13 AA had been deleted (_FLAG_Srr1-BRΔlatch). As shown Figure 8A, this mutation abolished the binding of the Srr1-BR. Moreover, untagged Srr1-BRΔlatch (100 µg/ml) failed to inhibit the binding of _FLAG_Srr1-BR binding to immobilized fibrinogen (data not shown). The Srr1-BR protein readily bound to hBMEC and this interaction was increased by preincubating hBMEC with fibrinogen (20 µg/ml) (Figure 8B). In contrast, the Srr1-BRΔlatch protein exhibited lower levels of binding to hBMEC compared with the Srr1-BR protein, which were not enhanced by fibrinogen. To exclude the possibility that this deletion had produced changes in the secondary structure of the protein that might account for the reduction in fibrinogen-binding activity, we analyzed Srr1-BR and Srr1-BRΔlatch proteins by circular dichroism (Fig. S6). The two proteins had a similar CD profile, with a maximum at less than 200 and a minimum at 216–218, resembling previously determined CD spectra for ClfA [29]. These results indicate that the Srr1-BR mediates Srr1 binding to fibrinogen, and that the C-terminal end of Srr1-BR contains a latch-like domain.

We next generated an isogenic variant of strain GBS NCTC 10/84 in which the latch-like domain of the Srr1-BR had been deleted. Of note, deletion of this region did not affect surface expression of Srr1 (Figure 8C). We then examined the impact of this mutation on GBS binding to fibrinogen and brain endothelium. As shown in Fig. 8D and E, deletion of the latch region significantly reduced GBS binding to fibrinogen and hBMEC, as compared with the parent strain. These results strongly suggest that GBS binding to fibrinogen is mediated by Srr1-BR via the “dock, lock, and latch” mechanism.

### Impact of Srr1 latch domain on GBS virulence and the development of meningitis

**Figure 9:**
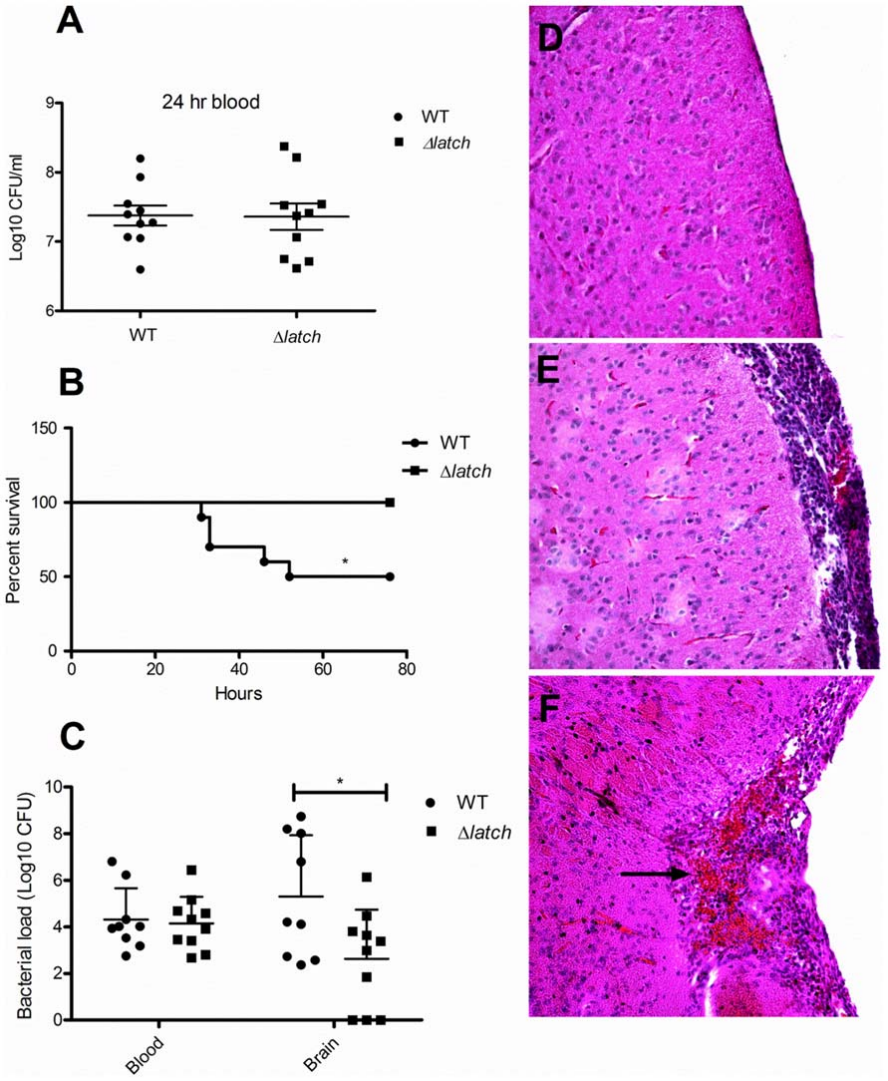
Impact of the latch-like domain on GBS virulence. Bacteria counts in the blood and brain of mice infected with GBS WT or Δl*atch* bacteria, determined 24 h post infection (A) or at the time of death (C). The horizontal lines denote the median number of bacteria in each group of 10 mice. (B) Kaplan-Meier survival curves of CD-1 male mice following i.v. infection with GBS NCTC 10/84 or Δlatch strains. (n = 10 per group). (D–F) Histopathology of representative brain tissues from mice infected with GBS*Δlatch* (D) and WT GBS (E, F). Note significant increase of meningeal thickness and neutrophil infiltration in WT infected brain tissue, but not in animals infected with the Δ*latch* variant.

To investigate the role of Srr1–mediated binding to fibrinogen in the pathogenesis of experimental meningitis, we compared the relative virulence of NCTC 10/84 with its isogenic latch-deficient variant. CD-1 mice were infected intravenously with either the WT or the Δ*latch* mutant strain. Twenty-four hours after challenge, the levels of GBS detected in the blood of each group were essentially identical (Figure 9A). Despite their initial similarities in establishing a high-grade bacteremia in the mouse, infection with the WT GBS strain resulted in significantly higher mortality (*p* = 0.017, Log Rank test). By 54 h, 50% of mice infected with NCTC10/84 had died. In contrast, all animals infected with GBSΔ*latch* were alive at 78 h (Figure 9B). At the time of death (or upon euthanasia at 78 h), blood and brain were harvested from each mouse for quantitative bacterial culture. Mice infected with the WT strain exhibited significantly higher final bacterial loads and penetrated into the brain more frequently than the Δ*latch* mutant (Figure 9C). Histologic examination of brain tissue from mice infected with the Δ*latch* mutant showed normal brain morphology with no signs of inflammation or injury (Figure 9D), whereas mice infected with WT GBS showed meningeal thickening, tissue destruction and neutrophil infiltration (Figure 9E and 9F).

## Discussion

The SRR proteins of GBS are thought to be important both for colonization of the female genital tract, and for the pathogenesis of invasive diseases, such as sepsis and meningitis. Expression of Srr1 has been shown to enhance the attachment of bacteria to vaginal and cervical epithelial cells *in vitro*, and to facilitate genital colonization in mice [30]. These interactions may be mediated in part by the binding of Srr1 to cytokeratin 4 on the surface of these epithelial cells. Studies *in vitro* indicate that the Srr1 interacts with cytokeratin 4 to promote bacterial attachment to the cell surface [14], [30]. However, binding can be blocked by sWGA, suggesting that the glycosylated serine-rich domains may also be involved in the interaction of Srr1 with cytokeratin 4 [14]. Strains expressing Srr1 are also more virulent in animal models of meningitis, as compared with their isogenic, *srr1*-deleted variants [7], [8]. Expression of Srr1 enhances GBS binding to hBMEC, which is likely to be an essential step for initiating central nervous system invasion and meningitis [7].

Our results now demonstrate that Srr1 promotes the adherence of GBS to human fibrinogen, and that this process is likely to be important for the pathogenesis of meningitis. Binding occurs via the interaction of Srr1-BR with the C-terminus of the fibrinogen Aα chain. This appears to be a specific event, requiring the entire Srr1-BR, and amino acids 283–410 of the Aα chain. Although Srr1 has limited primary sequence similarity to other known fibrinogen binding proteins, our secondary structure analyses indicate that Srr1-BR is likely to have a conformation resembling that of ClfA and possibly other related proteins, such as SdrG of *Staphylococcus epidermidis*. These and a number of other Gram-positive bacterial adhesins are thought to bind fibrinogen through a “dock, lock, and latch” (DLL) mechanism [24]–[26], as described above. Deletion of the predicted latch-like domain of Srr1 significantly reduced fibrinogen binding by the recombinant protein, as well as by bacteria, suggesting that Srr1 binding occurred by a comparable mechanism. If so, this would be the first example of a streptococcal DLL adhesin. Notwithstanding these similarities, there are some notable differences between Srr1 and its staphylococcal counterparts. For example, while Srr1 binds the Aα chain of fibrinogen, ClfA recognizes the C-terminus of the γ chain, and SdrG binds the N-terminus of the β chain [24], [25], [27]. Although both Srr1 and ClfB bind the C-terminus of the Aα chain, their binding sites on fibrinogen appear to differ [27], [28], [31]. A recombinant peptide representing the Aα chain binding site for ClfB (AA283–347) did not inhibit Srr1-BR binding to fibrinogen (Figure S7). Conversely, a peptide containing Aα chain residues 348–410 effectively blocked Srr1-BR binding, but no effect on ClfB binding to fibrinogen. These findings suggest that, while the binding of Srr1 to the Aα chain has some features in common with ClfB, the interactions of these adhesins with fibrinogen must also differ significantly. Further understanding of the precise basis for Srr1 binding to fibrinogen, and whether it occurs via a DLL mechanism, will require solution of its crystal structure.

Srr1 binding to fibrinogen was also important for the attachment of GBS to hBMEC *in vitro*. Binding of GBS to brain endothelium was reduced by deletion of the putative latch domain of Srr1, and was significantly enhanced by adding human fibrinogen, at concentrations (20 µg/ml) well within those found in whole blood (2–4 mg/ml) [32]. These findings indicate that the Srr1-fibrinogen binding is a relevant process for CNS invasion, and indeed we found that in mice with experimental meningitis, the latch deletion was also associated with significantly reduced levels of bacteria, mortality, and inflammation within the CNS. Of note, levels of the bacteria within the bloodstream were not altered by the above mutation, further indicating that the virulence properties associated with Srr1 and fibrinogen binding are specific to CNS infection.

FbsA and FbsB are two additional fibrinogen binding proteins of GBS that have been characterized [33], [34]. These proteins appear to be structurally unrelated to Srr1 or other known fibrinogen binding proteins. FbsA and FbsB can bind fibrinogen directly *in vitro*, although their binding sites on fibrinogen have not been identified. FbsA can also enhance the attachment of GBS to hBMEC [35]. However, FbsA alone is not sufficient for cell invasion, but appears to require FbsB for this process [36]. The contribution of FbsA and FbsB, and their interactions with fibrinogen to virulence is not well-defined. Neither protein has been examined for its role in the pathogenesis of meningitis. Deletion of *fbsA* was associated with decreased virulence in an animal model of septic arthritis and septicemia [37]. However, neither active nor passive immunization with FbsA or FbsA-specific antibodies resulted in protection against subsequent infection [37], suggesting that the virulence properties of FbsA may be unrelated to fibrinogen binding. Two other GBS proteins (the fibronectin binding protein Fib and a predicted ABC transport protein SAG0242) have been shown to bind fibrinogen, but neither the mechanisms for protein binding, nor the biologic importance of these interactions, have been addressed [33].

In summary, our results show that Srr1 mediates the binding of GBS to fibrinogen, and that this interaction is likely to occur via a DLL-like mechanism, involving the C-terminus of the fibrinogen Aα chain. It is the first streptococcal adhesin for which this type of binding has been identified, indicating that DLL binding may be a generalized mechanism for attachment by Gram-positive organisms. In addition, Srr1-fibrinogen binding appears to be important for the adherence to brain endothelium and the development of meningitis Given that Srr1 or its homolog Srr2 appear to be expressed by most clinical isolates of GBS, this interaction may prove to be a promising candidate for novel therapies targeting bacterial virulence.

## Materials and Methods

### Ethics statement

This study was carried out in strict accordance with the recommendations in the Guide for the Care and Use of Laboratory Animals of the National Institutes of Health. The protocol was approved by the Institutional Animal Care and Use Committee of San Diego State University (Animal Welfare Assurance Number: A3728-01). All efforts were made to minimize suffering of animals employed in this study.

### Reagents

Purified human fibrinogen was obtained from Haematologic Technologies. Rabbit anti-fibrinogen IgG was purchased from Aniara. Rabbit anti-Srr1 IgG was generated using purified Srr1-BR protein (NeoPeptide).

### Strains and growth conditions

The bacteria and plasmids used in this study are listed in Table S1 and S2. *S. agalatiae* strains were grown in Todd-Hewitt broth (Difco) supplemented with 0.5% yeast extract (THY). All mutant strains grow comparably well *in vitro* (data not shown). *Escherichia coli* strains DH5α, BL21 and BL21(DE3) were grown at 37°C under aeration in Luria broth (LB; Difco). Appropriate concentrations of antibiotics were added to the media, as required.

### Cloning and expression of Srr1-BR

Genomic DNA was isolated from GBS NCTC 10/84, using Wizard Genomic DNA purification kits (Promega), according to the manufacturer's instructions. PCR products were purified, digested, and ligated into pET28_FLAG_ to express FLAG-tagged versions of Srr1-BR (amino acids [AA] 303–641), the amino terminus of Srr1-BR (AA 303–479), the carboxy terminus of Srr1-BR (AA480–641) or the latch deletion of Srr1-BR (AA 303–628). Untagged Srr1-BR and Srr1-BRΔlatch were cloned into pET22b(+) (Novagen). The plasmids were then introduced to *E. coli* BL21(DE3) for over-expression. Proteins were purified by either Ni-NTA (Promega) or anti-FLAG M2 agarose affinity chromatography (Sigma-Aldrich), according to the manufacturers' instructions.

### Cloning and expression of fibrinogen chains

cDNAs encoding the Aα-, Bβ- and γ-chains of human fibrinogen were generously provided by Professor Susan Lord (University of North Carolina at Chapel Hill) [38]–[40]. The full length and truncated forms of chains were amplified and cloned into pMAL-C2X (New England Laboratory) to express MalE-tagged versions of the chains. Plasmids were then introduced to *E. coli* BL21 by transformation. All recombinant proteins were purified by affinity chromatography with amylose resin, according to the manufacturer's instructions (New England Biolabs).

### Analysis of Srr1-BR binding to fibrinogen by Far Western blotting

Purified human fibrinogen and recombinant fibrinogen chains were separated by electrophoresis through 4–12% NuPAGE Tris-Acetate gels (Invitrogen) and transferred onto nitrocellulose membranes. The membranes were treated with casein-based blocking solution (Western Blocking Reagent; Roche) at room temperature, and then incubated for 1 h with FLAG-tagged Srr1-BR (0.5 µM) suspended in PBS-0.05% Tween 20 (PBS-T). The membranes were then washed three times for 15 min in PBS-T, and bound proteins were detected with mouse anti-FLAG antibody (Sigma-Aldrich).

### Analysis of Srr1-BR binding to fibrinogen by enzyme linked immunosorbent assay (ELISA)

Purified fibrinogen (0.1 µM) was immobilized in 96-well microtiter dishes by overnight incubation at 4°C. The wells were washed twice with PBS and blocked with 300 µl of a casein-based blocking solution for 1 h at room temperature [41], [42]. The plates were washed three times with PBS-T, and _FLAG_Srr1-BR, _FLAG_Srr1-BR-N, _FLAG_Srr1-BR-C or _FLAG_Srr1-BRΔlatch in PBS-T was added over a range of concentrations. The plates were then incubated for 1 h at 37°C. Unbound protein was removed by washing with PBS-T, and the plates were incubated with mouse anti-FLAG antibodies diluted 1∶4000 in PBS-T for 1 h at 37°C. Wells were washed and incubated with HRP-conjugated rabbit anti-mouse IgG diluted 1∶5000 in PBS-T for 1 h at 37°C. The dissociation constant *K*_D_ for Srr1 binding was calculated using Prism software v. 4.0 (GraphPad).

For inhibition assays, the wells containing immobilized with fibrinogen (0.1 µM) were pretreated with rabbit anti-fibrinogen or rabbit IgG for 30 min, followed by washing to remove unbound antibody prior to the addition of _FLAG_Srr1-BR. In addition, _FLAG_Srr1-BR was coincubated with anti-Srr1 IgG or purified untagged Srr1-BR proteins on the wells immobilized with fibrinogen. After washing out unbound proteins, bound _FLAG_Srr1-BR was then assessed as described above.

### Fluorescent microscopy

hBMEC were fixed with 4% paraformaldehyde and fibrinogen was stained with rabbit anti-fibrinogen IgG (1∶1000) and Alexa Fluor 488 conjugated goat anti-rabbit IgG (Invitrogen). Coverslips were mounted on glass slides using Vectashield (Vector labs) and visualized with a confocal laser scanning microscope (Leica Microsystems).

### Binding of GBS to immobilized fibrinogen

Overnight cultures of GBS were harvested by centrifugation and adjusted to a concentration of 10^6^ CFU/ml in PBS. Purified fibrinogen (0.1 µM) was immobilized in 96-well microtiter plates as described above, and then incubated with 100 µl of GBS suspension for 30 min at 37°C. The wells were then washed to remove unbound bacteria, and then treated with 100 µl of trypsin (2.5 mg/ml) for 10 min at 37°C to release the attached bacteria. The number of bound bacteria was determined by plating serial dilutions of the recovered bacteria onto THB agar plates as previously described [41].

### Cell lines and infection assay

The human brain microvascular endothelial cell line (hBMEC) was developed and kindly provided by Kwang Sik Kim (Johns Hopkins University) [43], [44] and cultured as previously described [45]. Bacterial adherence assays were performed as described [46]. In brief, bacteria were grown to mid-log phase and then added to confluent hBMEC monolayers at a multiplicity of infection (MOI) of 0.1. After 30 min incubation, monolayers were washed 6 times with PBS to remove non-adherent bacteria, lysed and plated on THB agar to enumerate the bacteria. Bacterial adherence was calculated as (recovered CFU/initial inoculum CFU)×100%. In indicated experiments exogenous fibrinogen (20 µg/ml) was added directly to bacteria and incubated 1.5 hours with rotation at 37°C prior to addition to hBMEC monolayers.

### Western blot and lectin blot analysis of GBS Srr1

GBS cell wall extracts were prepared by treatment with spheroplasting buffer (500 units/ml mutanolysin, 20 mM Tris, 10 mM MgCl_2_·6H_2_O, and 0.5 M raphinose), as described previously [47], [48]. Proteins were separated by SDS-PAGE with 3–8% Tris-Acetate gels (Invitrogen) under reducing conditions and then were transferred to nitrocellulose membranes. After blocking with casein based blocking reagent (Roche), the membranes incubated with either 1) anti-Srr1-BR IgG (1∶3000) following by incubation with anti-rabbit IgG (1∶10,000); or 2) biotin conjugated wheat germ agglutinin (WGA; Vector Labs) (0.2 µg/ml) followed by incubation with HRP conjugated streptavidin (0.2 µg/ml).

### Mouse model of meningitis

A murine model of hematogenous GBS meningitis has been described previously [46]. Outbred 6- to 8-week old male CD-1 mice (Charles River Laboratories; 10 mice per group) were injected via the tail vein with 5×10^7^ CFU WT GBS (NCTC 10/84) or GBSΔ*latch* mutant. At 24 h post GBS injection, blood was collected via tail vein (20 µl) and plated on THB agar to determine the bacterial load in the bloodstream. Mouse survival was accessed over time. At the time of death, or at 78 h post infection, blood and brain tissue were collected aseptically from mice after euthanasia. Bacterial counts were in blood and tissue homogenates were determined by plating serial 10-fold dilutions on THB agar. Brain sections were also embedded in paraffin and stained with hematoxylin and eosin (H&E).

### Bioinformatic analysis

Amino acid similarity was compared using PSI-BLAST and secondary structure was determined by the prediction servers (PHYRE and HHPRED) [19], [49], [50].

### Data analysis

Data were expressed as means ± standard deviations and were compared for statistical significance by the unpaired *t* test.

## Supporting Information

Figure S1 **Inhibition of GBS NCTC10/84 binding to fibrinogen by anti-Srr1 IgG.** 10^6^ CFU of GBS strain NCTC 10/84 were incubated with anti-Srr1 IgG or rabbit IgG in 96 well plates coated with fibrinogen. Values represent percent of WT GBS binding to wells treated with fibrinogen. * = P<0.01.
(TIF)

Figure S2 **Complementation of the** ***srr1*** **mutation** ***in trans*** **restores fibrinogen binding by NCTC 10/84 Δ** ***srr1*** **.** A. Fibrinogen binding by NCTC (WT), its Δ*srr1* mutant, and the mutant complemented with pDE123 alone or the vector encoding *srr*1. The *srr*1 mutant complemented with encoding *srr*1 gene demonstrated significantly greater levels of binding than Δ*srr*1 and Δ*srr*1 with pDE123 control vector. * = P<0.01. B. Expression of Srr1 on the cell surface of complementation strain. Isolated cell wall proteins were probed by Western blotting with WGA lectin. Note lower level of Srr1 expression on the complementation strain.
(TIF)

Figure S3 **Binding of Srr1-BR to recombinant MalE-Aα chain.** Recombinant MalE-Aα, Bβ, and γ chains were separated by SDS-PAGE and stained with Coomassie blue (left) or transferred to nitrocellulose, and probed with _FLAG_Srr1-BR (right).
(TIF)

Figure S4 **Inhibition of GBS COH31 binding to fibrinogen by purified MalE:Aα_283–410_.** 10^6^ CFU of WT or Δ*srr1* GBS were incubated with MalE:Aα_283–410_ in 96 well plates coated with fibrinogen. Values represent percent of WT GBS binding. * = P<0.01.
(TIF)

Figure S5 **Sequence analysis of Srr1 and SdrG.** Sequence alignment of the Srr1-BR with corresponding regions of SdrG. Red, blue and black letters represent charged, polar and hydrophobic residues, respectively. Blue box and red box indicates TYTFTDYVD-like “latching cleft” and “latch” motif respectively.
(TIF)

Figure S6 **Analysis of secondary-structure by far-UV CD spectroscopy.**
(TIF)

Figure S7 **Inhibition of ClfB-N2N3 and Srr1-BR binding to immobilized fibrinogen with MalE fused fibrinogen Aα_283–347_ (A) or Aα_348–410_ (B).**
(TIF)

Table S1 **Bacterial strains.**
(DOC)

Table S2 **Plasmids.**
(DOCX)

Table S3 **Latching cleft and latch domains within the binding regions of fibrinogen binding proteins.**
(DOCX)
