# Supplementary figures and images for: Binding of Glycoprotein Srr1 of Streptococcus agalactiae to Fibrinogen Promotes Attachment to Brain Endothelium and the Development of Meningitis
Source: PLoS Pathog. 2012 Oct 4;8(10):e1002947. doi: 10.1371/journal.ppat.1002947 (PMC3464228; doi:10.1371/journal.ppat.1002947)

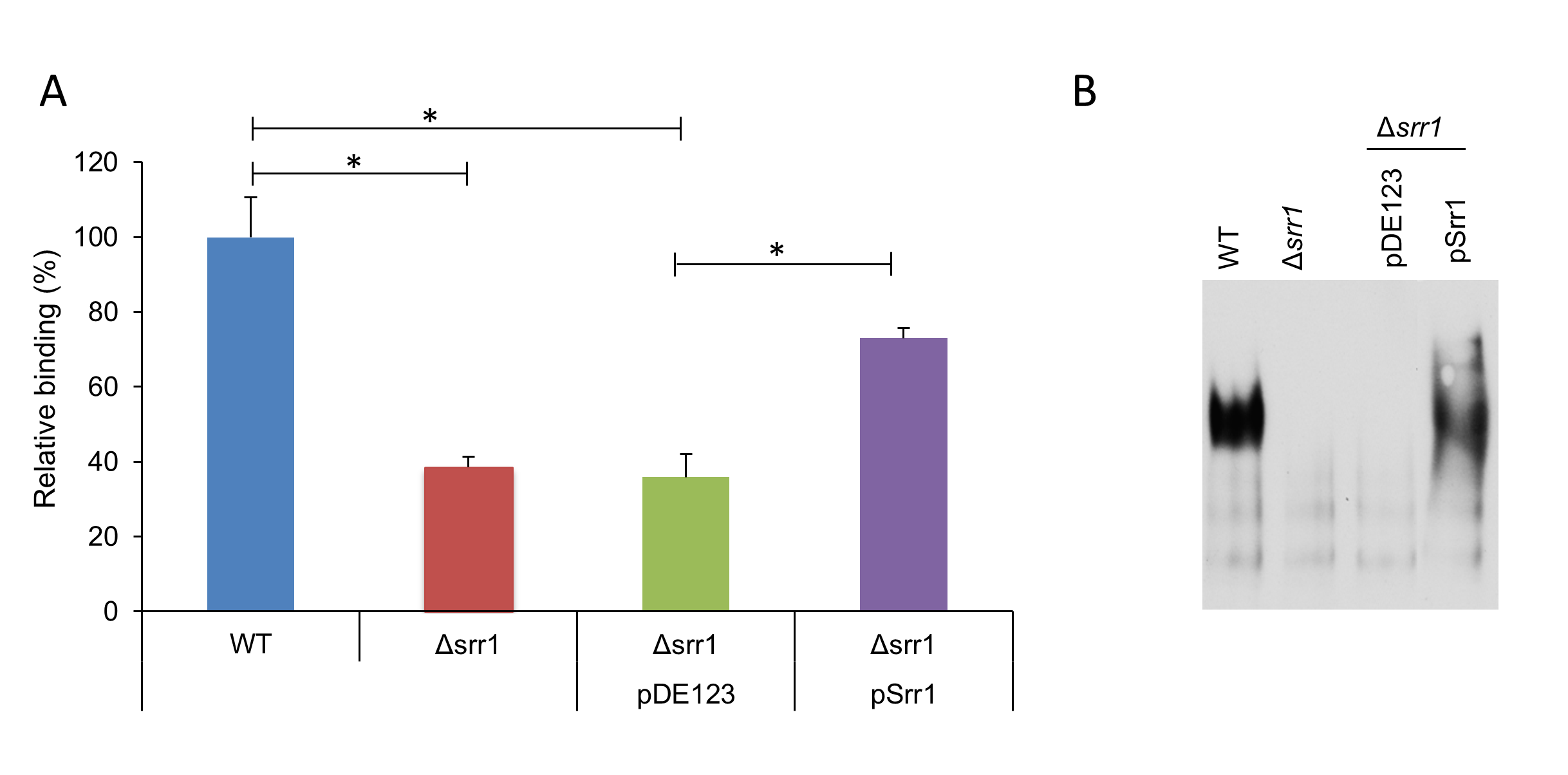

Supplement: Figure S2 — Complementation of the srr1 mutation in trans restores fibrinogen binding by NCTC 10/84 Δ srr1 . A. Fibrinogen binding by NCTC (WT), its Δsrr1 mutant, and the mutant complemented with pDE123 alone or the vector encoding srr1. The srr1 mutant complemented with encoding srr1 gene demonstrated significantly greater levels of binding than Δsrr1 and Δsrr1 with pDE123 control vector. * = P<0.01. B. Expression of Srr1 on the cell surface of complementation strain. Isolated cell wall proteins were probed by Western blotting with WGA lectin. Note lower level of Srr1 expression on the complementation strain. (TIF) [file ppat.1002947.s002.tif]

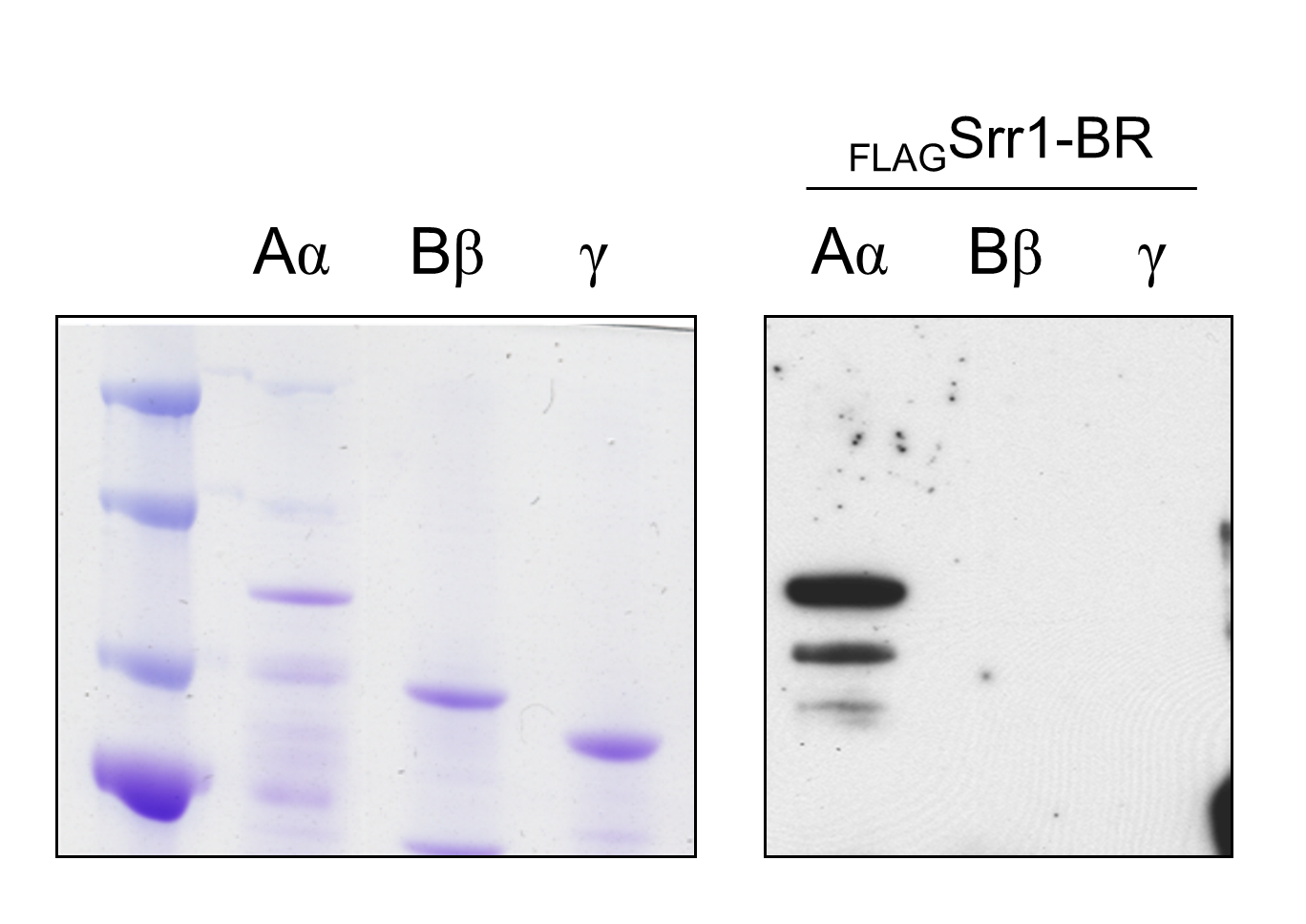

Supplement: Figure S3 — Binding of Srr1-BR to recombinant MalE-Aα chain. Recombinant MalE-Aα, Bβ, and γ chains were separated by SDS-PAGE and stained with Coomassie blue (left) or transferred to nitrocellulose, and probed with FLAGSrr1-BR (right). (TIF) [file ppat.1002947.s003.tif]

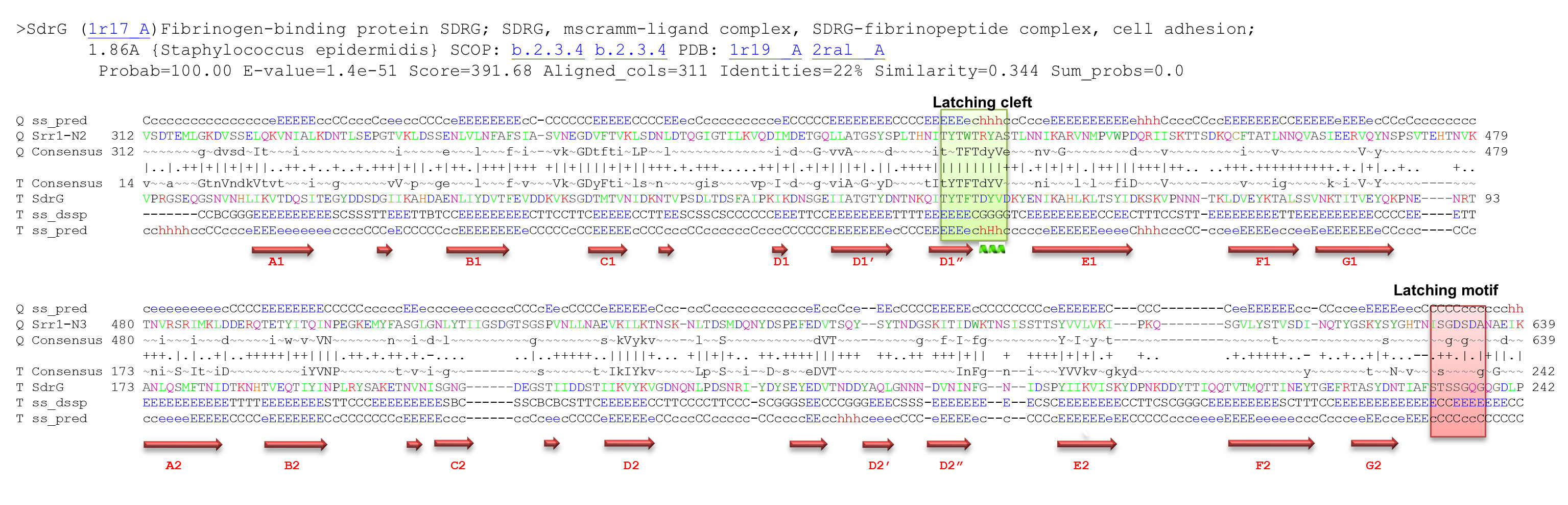

Supplement: Figure S5 — Sequence analysis of Srr1 and SdrG. Sequence alignment of the Srr1-BR with corresponding regions of SdrG. Red, blue and black letters represent charged, polar and hydrophobic residues, respectively. Blue box and red box indicates TYTFTDYVD-like “latching cleft” and “latch” motif respectively. (TIF) [file ppat.1002947.s005.tif]

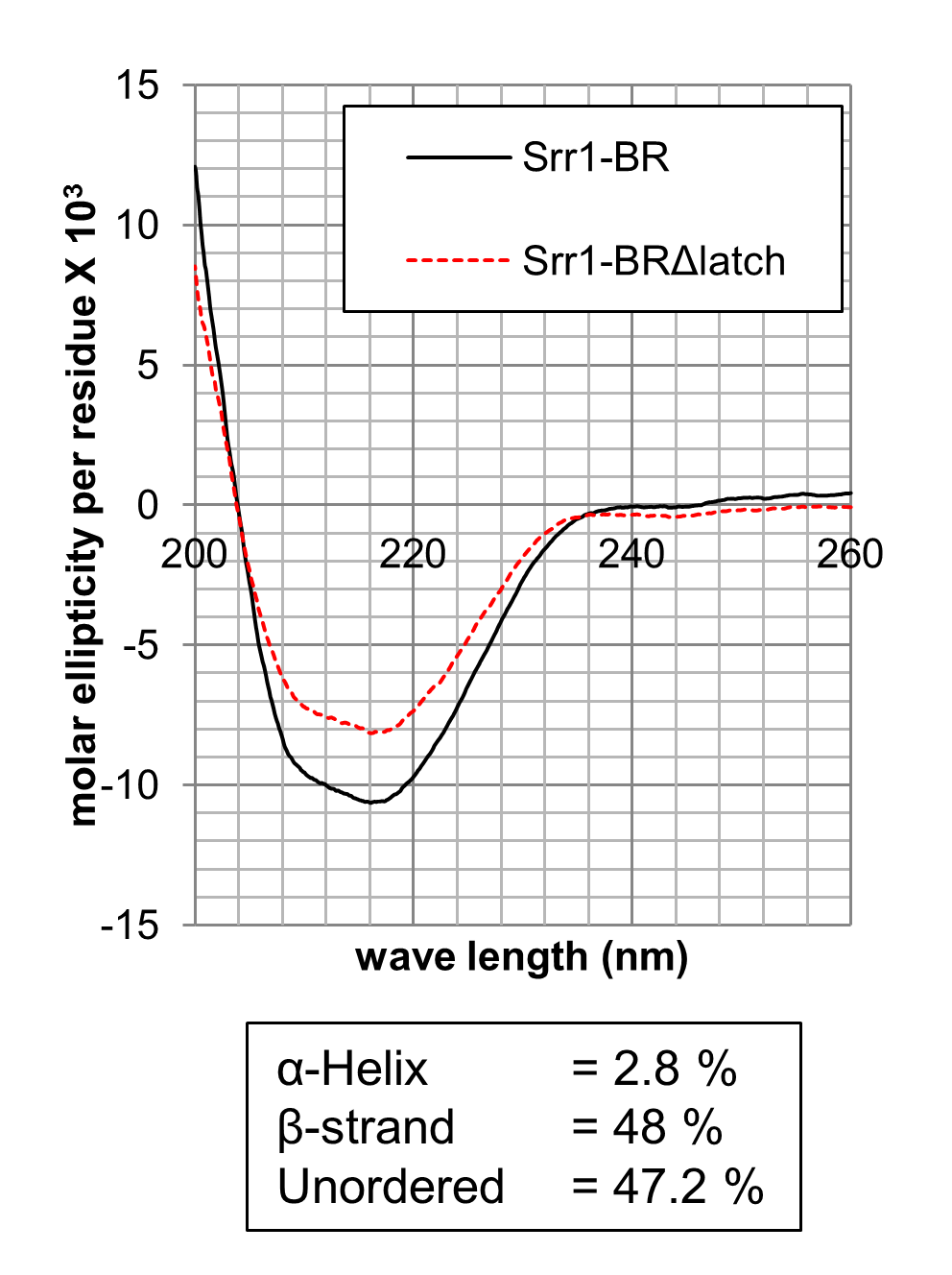

Supplement: Figure S6 — Analysis of secondary-structure by far-UV CD spectroscopy. (TIF) [file ppat.1002947.s006.tif]

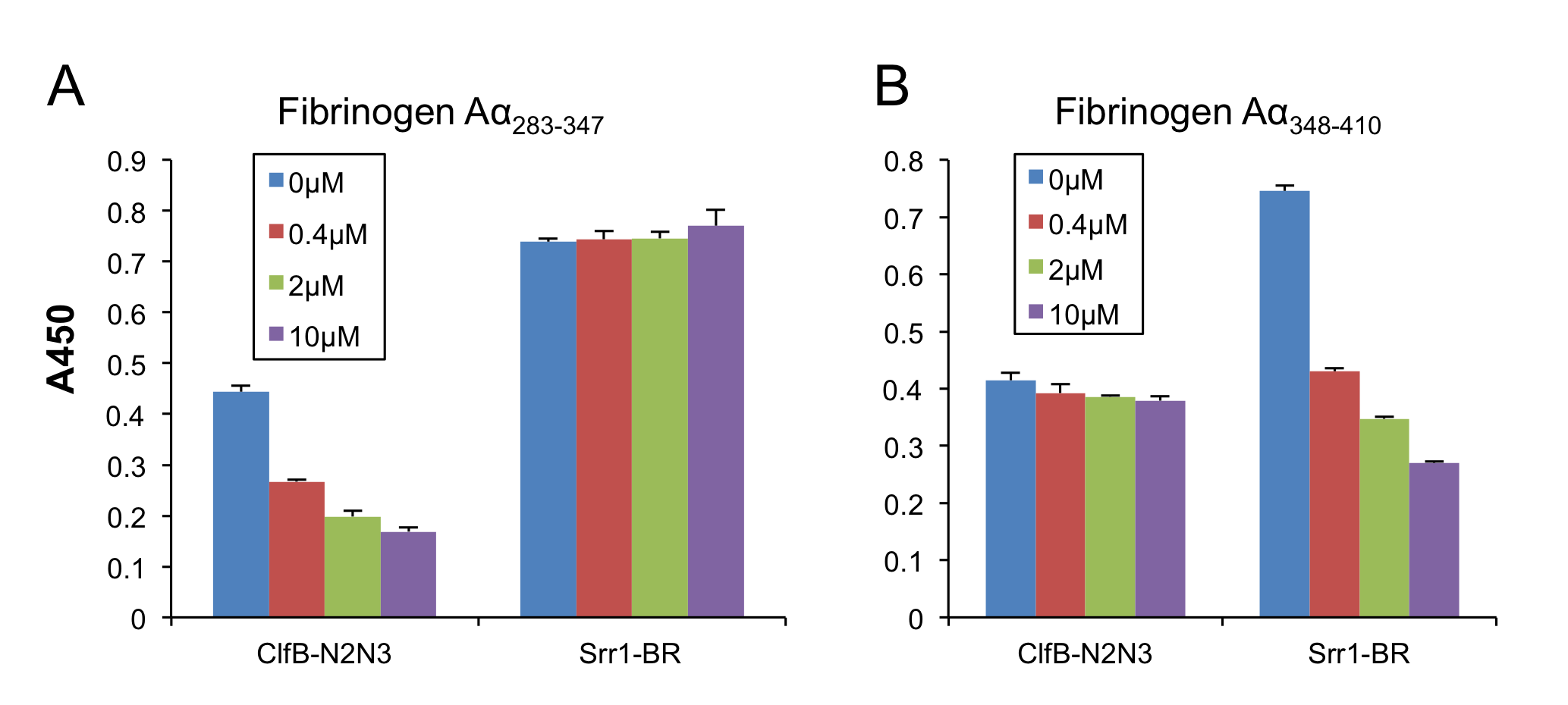

Supplement: Figure S7 — Inhibition of ClfB-N2N3 and Srr1-BR binding to immobilized fibrinogen with MalE fused fibrinogen Aα283–347 (A) or Aα348–410 (B). (TIF) [file ppat.1002947.s007.tif]
