# Supplementary material for: Binding of Glycoprotein Srr1 of Streptococcus agalactiae to Fibrinogen Promotes Attachment to Brain Endothelium and the Development of Meningitis
Source: PLoS Pathog. 2012 Oct 4;8(10):e1002947. doi: 10.1371/journal.ppat.1002947 (PMC3464228; doi:10.1371/journal.ppat.1002947)
Supplement: Table S1 — Bacterial strains. (DOC) [file ppat.1002947.s008.doc]

**Table S1. Strains**

| **Strain or plasmid** | **Genotype or description*^a^*** | | | | **Source** | |
| --- | --- | --- | --- | --- | --- | --- |
| *Escherichia coli* | | | |  | | |
| DH5α | F^-^r^-^m^+^Ø80d*lacZ*∆M15 | | | Gibco BRL | | |
| BL21 (DE3) | expression host, inducible T7 RNA polymerase | | | Novagen | | |
| *Streptococcus agalactiae* | | | |  | | |
| COH31 | | Serotype III, Clinical isolate | |  | | |
| PS954 | | COH31∆*srr*1, Cm^R^ | | this study | | |
| NCTC 10/84 | | Serotype V, Clinical isolate | |  | | |
| PS2645 | | NCTC 10/84∆*srr*1, Cm^R^ | | [1] | | |
| PS2844 | | NCTC 10/84∆latch, Erm^R^ | | this study | | |
| PS2710 | | PS2645/pDE123 | | this study | | |
| PS2646 | | PS2645/pDE123-Srr1 | | [1] | | |
| H36B | | Serotype Ib, Clinical isolate | |  | | |
| PS2648 | | H36B∆*srr*1, Cm^R^ | | this study | | |
| 515 | | Serotype Ia, Clinical isolate | |  | | |
| PS2643 | | 515∆*srr*1, Cm^R^ | | this study | | |
| D136C | | Serotype III, Clinical isolate | | | |  |
| NCTC 1/82 | | Serotype IV, Clinical isolate | | | |  |
| 2603 V/R | | Serotype V, Clinical isolate | | | |  |
| A909 | | Serotype Ia, Clinical isolate | | | |  |
| NEM316 | | | Serotype III, Clinical isolate | | |  |

^a^ Erm^R^, erythromycin resistance; Cm^R^, chloramphenicol resistance

**REFERENCES**

1. van Sorge NM, Quach D, Gurney MA, Sullam PM, Nizet V, et al. (2009) The group B streptococcal serine-rich repeat 1 glycoprotein mediates penetration of the blood-brain barrier. J Infect Dis 199: 1479-1487.
