# Supplementary material for: Binding of Glycoprotein Srr1 of Streptococcus agalactiae to Fibrinogen Promotes Attachment to Brain Endothelium and the Development of Meningitis
Source: PLoS Pathog. 2012 Oct 4;8(10):e1002947. doi: 10.1371/journal.ppat.1002947 (PMC3464228; doi:10.1371/journal.ppat.1002947)
Supplement: Table S2 — Plasmids. (DOCX) [file ppat.1002947.s009.docx]

**Table S2. Plasmids**

| **Plasmid** | **Description** | **Source** | |
| --- | --- | --- | --- |
| pDE123 | Streptococcal shuttle vector, Erm^R^ | | this study |
| pDE123-*srr1* | vector for expression of Srr1, Erm^R^ | | [1] |
| pET28a+ | expression vector, Kan^R^ | | Novagen |
| pET28_FLAG_ | expression vector with FLAG-tag, Kan^R^ | |  |
| pET28-Srr1-BR | vector for expression of Srr1N2N3, Kan^R^ | | this study |
| pET28_FLAG_Srr1-BR_303-641_ | vector for expression of FLAG-tagged Srr1_303-641_ | | this study |
| pET28_FLAG_Srr1-BR_303-479_ | vector for expression of FLAG-tagged Srr1_303-479_ | | this study |
| pET28_FLAG_Srr1-BR_480-641_ | vector for expression of FLAG-tagged Srr1_480-641_ | | this study |
| pET28_FLAG_Srr1-BRΔlatch | vector for expression of FLAG-tagged Srr1_303-627_ | | this study |
| pSET-5S | Streptococcal thermosensitive suicide vector,Cm^R^ | | [2] |
| pSET-5S-*srr*1KO | vector for deletion of *srr1* gene, Cm^R^ | | this study |
| pSET-5S-*latch*KO | vector for deletion of latch domain, Cm^R^ Erm^R^ | | this study |
| pMAL-C2X | expression vector with MalE fusion protein | | NEB |
| pMal-Aα | vector for expression of MalE-tagged Aα chain | | [3] |
| pMal-Bβ | vector for expression of MalE-tagged Bβ chain | | [3] |
| pMal-γ | vector for expression of MalE-tagged γ chain | | [3] |
| pMal-Aα_1-197_ | vector for expression of MBP-tagged Aα variant | | this study |
| pMal-Aα_198-610_ | vector for expression of MBP-tagged Aα variant | | this study |
| pMal-Aα_198-282_ | vector for expression of MBP-tagged Aα variant | | this study |
| pMal-Aα_283-410_ | vector for expression of MBP-tagged Aα variant | | this study |
| pMal-Aα_1(198-282+411-610)_ | vector for expression of MBP-tagged Aα variant | | this study |

**REFERENCES**

1. van Sorge NM, Quach D, Gurney MA, Sullam PM, Nizet V, et al. (2009) The group B streptococcal serine-rich repeat 1 glycoprotein mediates penetration of the blood-brain barrier. J Infect Dis 199: 1479-1487.

2. Takamatsu D, Osaki M, Sekizaki T (2001) Thermosensitive suicide vectors for gene replacement in *Streptococcus suis*. Plasmid 46: 140-148.

3. Seo HS, Sullam PM (2011) Characterization of the fibrinogen binding domain of bacteriophage lysin from *Streptococcus mitis*. Infect Immun 79: 3518-3526.
