# Supplementary material for: Binding of Glycoprotein Srr1 of Streptococcus agalactiae to Fibrinogen Promotes Attachment to Brain Endothelium and the Development of Meningitis
Source: PLoS Pathog. 2012 Oct 4;8(10):e1002947. doi: 10.1371/journal.ppat.1002947 (PMC3464228; doi:10.1371/journal.ppat.1002947)
Supplement: Table S3 — Latching cleft and latch domains within the binding regions of fibrinogen binding proteins. (DOCX) [file ppat.1002947.s010.docx]

**Table S3. Latching cleft and latch domains within the binding regions of fibrinogen binding proteins**

| MSCRAMM  (Accession number) | Latching cleft | Number of residues between latching cleft and latch | Latch  motif | Organism |
| --- | --- | --- | --- | --- |
| SdrG (AAF72510) | TYTFTDYVD | 207 | SSGQGQG | *S. epidermidis* |
| SdrF (AAF72509) | TYTFTNYVD | 200 | GSSTAQG | *S. epidermidis* |
| ClfA (CAA79304) | IYTFTDYVN | 207 | GSGSGDG | *S. aureus* |
| ClfB (CAA12115) | TFVFTDYVN | 204 | GGGSADG | *S. aureus* |
| FnbpA (CAA65106) | RYTFTNDIE | 210 | NKANGNE | *S. aureus* |
| FnbpB (CAA44726) | RYTFKEYVQ | 204 | NNAQGDG | *S. aureus* |
| Srr1 (NP735966) | TYTWTRYAS | 207 | GDSDANA | *S. agalactiae* |
